# Prevalence of Depression among Type-II Diabetic Patients Attending the Diabetic Clinic at Arar National Guard Primary Health Care Center, Saudi Arabia

**DOI:** 10.1155/2020/9174818

**Published:** 2020-06-19

**Authors:** Norah Muqbil Alhunayni, Amal Elwan Mohamed, Sabry Mohamed Hammad

**Affiliations:** ^1^Resident of Family Medicine, Saudi Board of Family Medicine at the Northern Borders Region, Saudi Arabia; ^2^Consultant of Public Health and Community Medicine, Faculty of Medicine, Zagazig University, Zagazig, Egypt; ^3^Consultant of Public Health and Community Medicine, Faculty of Medicine, Mansoura University, Mansoura, Egypt

## Abstract

**Background:**

Depression is a common comorbidity in type-II diabetic patients, which if undiagnosed leads to poor clinical outcomes.

**Objectives:**

To determine the prevalence and risk factors of depression among type-II diabetic patients attending the National Guard Diabetic Clinic in Arar city. *Subjects and methods*. This cross-sectional study included every third type-II diabetic patient attending the National Guard Primary Health Care Center between the 1^st^ of January and 31^st^ of March 2019. Participants were interviewed using the Patient Health Questionnaire 9 (PHQ-9). Patients who scored ≥5 were considered to have depression. Chi-square test (*χ*^2^) was used to compare differences between categorical variables. *P* ≤ 0.05 was considered statistically significant.

**Results:**

Of the total 422 diabetic patients approached for this study, 397 provided complete responses (94% response rate). Of these, 37% had depression: 23% mild, 9% moderate, and 5% severe. Diabetic patients with low education, poor income, and long duration of diabetes mellitus were found to be at higher risk of depression. Poor compliance with physical activities, diet regimen, family history of DM, and the presence of complications was also significantly associated with depression. On logistic regression analysis, low family income, duration of DM, poor compliance to physical activity, and presence of complications as neuropathy or libido were the main predictors of depression in diabetic patients.

**Conclusion:**

More than one-third of type-II diabetic patients had depression. Regular screening of type-II diabetic patients for depression is a necessity, as it can affect the clinical outcome.

## 1. Introduction

Diabetes (DM) represents a challenging set of biopsychosocial conditions affecting both the patients and their families. Therefore, the patients are advised to use medications and adopt multiple self-care behaviors to achieve glycemic control [1]. Social and environmental factors affecting self-care have a considerable burden on patients and their families. This burden may lead to depression. Diabetic patients suffering from depression may also have poor health behaviors, low income, stigma, and lack of social support [2]. Diabetes is increasing in Saudi Arabia. The prevalence of type-II diabetes in Saudi Arabia is 32.8%. The predicted prevalence of DM will be 35.37% in 2020 and increase to 40.37% in 2025 and 45.36% in the year 2030 [3].

Depression is very common among patients with type 2 diabetes, and it is related to diabetes outcomes. In Saudi Arabia, a recent study in Arar city found that the prevalence of depression among diabetic patients was 37.4% [4]. Also, in a study in Eastern Province, the prevalence of depression among type 2 diabetic patients was 49.6% [5]. In Qassim, a study found that 34.8% of the study participants experienced depression [6].

Therefore, depression is two times higher in diabetic patients; the majority of the cases remain undiagnosed [7]. Comorbid diabetes and depression are major clinical challenges as each one is worsened by the other. The psychological burden from living with diabetes may lead to depression [8, 9]. Depression is related to poor compliance with diabetes self-care, including following the dietary regimen, medication adherence, and blood glucose monitoring, resulting in worse whole clinical outcomes [10].

In consequence, the course of depression in diabetic patients is chronic and severe. More than 80% of patients with diabetes and depression will suffer a relapse of depressive symptoms over five years. In addition to that, depression may be undiagnosed and untreated in almost two-thirds of patients with both conditions [11]. Moreover, the presence of depression in a person with diabetes may lead to a 36.8% increase in coronary heart disease and a 47.9% increase in cardiovascular mortality [12].

Stigma may lead to avoid seeking psychiatric treatment associated with reporting an embarrassing problem or misinterpretation of mental illness especially in Saudi diabetics [13].

Up to our knowledge, only one study has been conducted in the Northern Borders region, KSA to assess the prevalence of stress, anxiety, and depression among diabetic patients aged ≥12 years. The study investigated only demographic risk factors. The researchers found that patient age, being unmarried, and low education was significantly related to depression. Also, the prevalence of depression among type 2 DM and other risk factors were not explored [4]. Therefore, the current study aimed to determine the prevalence of depression among type-II diabetic patients in Arar city and to identify its associated risk factors.

## 2. Participants and Method

This cross-sectional study included type-II diabetic patients attending the diabetic clinic at Arar National Guard Primary Health Care Centre, Saudi Arabia, between the 1^st^ of January and 31^st^ of March 2019.

On reviewing the appointment list of the diabetic clinic in the National Guard Primary Health Care Center, the average daily attendance was 21 patients. Seven patients per working day were selected by a systematic random sampling technique after the selection of the first one by random choice. So, every 3rd diabetic was included in the study. Type 2 DM patients aged <18 years, pregnant, or diagnosed with psychiatric disorders were excluded from this study.

The sample size was estimated according to the sample size equation:

*n* = *z*_*α*_^2^*p* (1 − *p*)/*d*^2^ [14]. *n* is the sample size, *p* is the expected prevalence of depression among patients of type 2 diabetes mellitus = 49.6% according to a previous study conducted in Eastern province, KSA [5]. *Zα* = 1.96 and *d* = 0.05. The minimum required sample was 384. After accounting for a 10% noncompliance rate and incomplete forms, the final sample size was calculated to be 422.

The data was collected by a personal interview with the participants using a predesigned questionnaire, including two sections. The first section inquires about socio-demographic data. The second section is the Arabic version of the Patient Health Questionnaire-9, which is a valid and reliable screening tool for detecting depression [15]. Depression was considered as mild, with a score from 5 to 9 and moderate with a score ranging from 10 to 14. A score above 14 was considered to have severe depression [16]. Data on microvascular complications (retinopathy, neuropathy, and nephropathy) was retrieved from patient records.

Data was coded and analyzed using statistical package for social sciences (SPSS Inc.) version 20. Qualitative data was presented as frequencies and percentages. A chi-square test for independence was used to detect relations between depression and its risk factors. *P* value ≤0.05 was considered statistically significant. Factors significantly associated with depression were analyzed with binary logistic regression using the default methods.

Informed consent was obtained from each participant verbally before the interview. No name was recorded on the questionnaires. Data was dealt with confidentiality. Ethical approval was obtained from the regional ethical committee at the Northern borders region (no 6/1440) at 12/11/2018.

## 3. Results

Of the total 422 diabetic patients approached for this study, 397 provided complete responses, giving a 94% response rate. Their mean age was 48.8 ± 7.7 years, and 55.9% were males.

**Figure 1:**
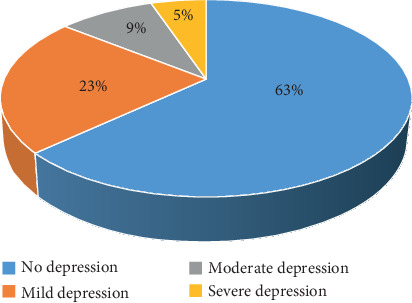
Percentage of depression among studied type 2 diabetic patients.

This study found that in these participants, 37% had depression (23% mild, 9% moderate, and 5% severe) (Figure 1). Further, it was found that patients with lesser than secondary education were twice likely to have depression than those with secondary education and above (OR = 2.05, CI: 1.34–3.13, *P* = 0.0007). Low family income was also associated with a significant increase in the risk of depression (OR = 3.78, CI: 1.4–10.2, *P* = 0.006). Other socio-demographic actors were not significantly related to depression (Table 1).

It was found that the risk of depression among type 2 diabetic patients increased significantly with the increase in the duration of disease (OR = 5.83, CI: 3.48–9.77, *P* = 0.0001). Patients on both oral hypoglycemic drugs and insulin were found to be more likely to have depression than among those on oral hypoglycemic drugs only (OR = 6.78, CI: 4.1–11.2, *P* = 0.0001). In contrast, compliance with diet and physical activity was significantly associated with a low risk of depression. Family history of DM, compliance with medications, regular foot examination, and daily blood sugar measurement were not significantly related to depression (Table 2).

The presence of complications (retinopathy, nephropathy, neuropathy, and libido) was also found to be significantly related to the risk of depression. Patients with a family history of depression were three times more likely to have depression than those with no family history (OR = 3.19, CI: 1.05–9.72, *P* = 0.03) (Table 2).

Table 3 shows that low family income, long duration of DM, poor compliance to physical activity, presence of neuropathy, and libido were significant predictors of depression among people with diabetes.

## 4. Discussion

Depression is a frequent comorbidity in diabetic patients. Therefore, this is a descriptive study that aimed to determine the prevalence of depression and its associated risk factors among type-II diabetic patients. The current study found that 37% of diabetic patients had depression; a result comparable with results of studies was conducted in Jazan city, KSA which reported a prevalence of depression among diabetic patients 37.6% [17] and 40.6% [18]. In the Western region, a recent study found that 33.8% of participants had depression [19]. In the Eastern province, KSA, a higher prevalence of depression (49.6%) among diabetic patients was reported [5]. The results of the current study are in agreement with figures reported by a Palestinian study (40%) [20]. However, studies were conducted in Qatar, 52.5% [21], Ethiopia (47%) [22] and Mexican (48.27%) [23] indicated a higher prevalence of depression among diabetics. The variations in the reported prevalence of depression in diabetic patients in different studies may be explained by different methodologies, screening tools, and culture. Depression is prevalent in diabetic patients. Owing to the presence of a link between depression and diabetes, but it is unclear. The link may be explained by shared parallel biological processes that involve hypothalamic pituitary adrenal access, insulin resistance, and circadian rhythm [24]. Moreover, life long endless demands of diabetes care such as eating carefully, monitoring blood glucose, monitoring of symptoms of low or very high blood glucose, exercising, and fears of complications affect the quality of life of diabetic patients.

Besides, the present study revealed that the risk of depression among diabetic patients increases significantly with low education and low income or low social support. These findings are consistent with that of studies conducted in Qassim, Jazan, and Jorden [6, 18, 25]. In Quebec, the incidence of depression was higher among low social class [26]. Association between depression and low education may be explained by the fact that educated people have better jobs and less likely to be depressed. The study also revealed an increased risk of depression among diabetics with increased duration of DM. This finding is similar to that reported by two studies done in the Eastern and Western regions, KSA [5, 19]. This may be explained by the increased risk of complications with longer duration of disease, which in turn increases the risk of depression.

Also, diabetics treated by both insulin and oral hypoglycemic drugs were six times more at risk of depression than those on oral hypoglycemic drugs only. Other studies also reported similar findings [27–29]. Although strict glycemic control should be achieved, patients receiving intensified treatment with insulin should be regularly screened for depression. Those patients that can be treated with drugs have improved glycemic control beside their antidepressant effect.

Moreover, the present study found that depressed patients had poor compliance with diet and physical activity. A study conducted in Jordon demonstrated a significant association between depression among diabetics and poor compliance to the diet [25]. Another study conducted in Spain found that low physical activity is associated with a higher risk of depression [28].

Also, diabetic patients suffering from complications of diabetes were found to be more liable to depression. This finding is consistent with other studies. A study conducted by Sachdeva et al., in Patiala, 2016, concluded a higher prevalence of depression in type 2 diabetic patients with retinopathy, neuropathy, and nephropathy compared to those without these complications [30]. Patients with retinopathy will have frequent visits to ophthalmologists and may need injections in the eye or laser treatment. Nephropathy is also an indication of renal impairment that may need dialysis. Dialysis increases the risk of depression and needs further adjustments of medications.

Similarly, The present study revealed a higher prevalence of depression among diabetic patients with neuropathy than those without neuropathy. This association is in agreement with that reported in Spain study [28]. In another study conducted in Italy, a similar finding was observed by D'Amato et al., who stated a higher prevalence of depression among diabetic patients with peripheral neuropathy than in those without peripheral neuropathy. They also stated that diabetic peripheral neuropathic pain is a more significant predictor of depression than other complications of diabetes [31]. This can be explained that although some medications are available for the management of neuropathy, they may not always be sufficient to control the distress caused by neuropathy, and feeling pain can lead to depression.

Regarding the impact of libido on the occurrence of depression among type2 diabetic patients, the present study found that those with libido were nearly six times more likely to have depression than among those without libido (OR = 5.71, 95% CI: 3.19–10.22, *P* < 0.0001). A similar result was observed in the study conducted in Poland, which concluded a positive correlation between sexual disorder in diabetics and the occurrence of depression [32].

To sum up, type 2 diabetes is considered a lifestyle disease. Although guidelines for DM require medication adherence, diet, and exercise regimen, the depressed diabetic is less likely to adhere to physical activity and dietary restriction. Depression leads to an unhealthy lifestyle as harmful eating habits, lack of regular physical exercise, smoking, and weight gain. Worsening in glycemic control could be an indication that a person is depressed.

Both diabetes and depression can decrease life satisfaction and need a comprehensive approach to management. Coping with both conditions requires collaborative treatment with psychotherapy including counseling by cognitive behavioral therapy, evidence-based pharmacotherapy, and lifestyle modification [33]. Pharmacotherapy based on selective serotonin reuptake inhibitors (SSRIs) may relieve depressive symptoms in patients with comorbid depression and diabetes [34].

## 5. Conclusion

The percentage of depression is high among studied type 2 diabetic patients DM is frequently accompanied by depression. Regular screening for depression, treating depressive symptoms in diabetic patients can improve glycemic control. Prompt initiation of treatment for this concomitant condition is a necessity to ensure a better outcome and satisfactory quality of life.

## 6. Limitations

The study is a cross-sectional study, and the causal relationship cannot be established due to the bidirectional relation between diabetes and depression. The study used PHQ-9, which is a screening tool. Also, microvascular complications of diabetes were only included as predictors for depression. The study included patients attending the National Guard diabetic clinic, so the results of the study cannot be generalized to all diabetic population. Moreover, the relation between glycemic control and the occurrence of depression was not assessed.

## Figures and Tables

**Table 1:** Depression among type-II diabetic patients (*n* = 397) in relation to their socio-demographic data.

Characteristic	Total no (%)	Depression no (%)	*P* value	Odds ratio	95% CI
Socio-demographic data					
Age					
<30	9 (2.3%)	4 (44.4%)		r	
30-39	35 (8.8%)	10 (28.6%)	0.43	0.50	(0.11-2.25)
40-49	161 (40.5%)	56 (34.8%)	0.74	0.77	(0.20-2.98)
50+	192 (48.4%)	77 (40.1%)	0.53	0.83	(0.21-3.21)
Sex					
Male	222 (55.9%)	77 (34.7%)		r	
Female	175 (44.1%)	70 (40%)	0.296	1.26	(0.83-1.89)
Marital status					
Single	26 (6.6%)	8 (30.8%)		r	
Married	261 (65.7%)	84 (32.2%)	0.89	1.07	(0.44-2.55)
Divorced & widow	110 (27.7%)	55 (50%)	0.076	2.25	(0.90-5.6)
Educational level					
Less than secondary	155 (39%)	69 (44.5%)		2.05	(1.34-3.13)
Secondary +	242 (61%)	68 (28.1%)	0.0007	r	
Income in relation to expenditure:					
Not enough	21 (5.3%)	10 (47.6%)	0.006	3.78	(1.40-10.19)
Enough	278 (70%)	118 (42.4%)	<.0001	3.07	(1.76-5.34)
Enough and save	98 (24.7%)	19 (19.4%)		r	
Smoking					
No	290 (73%)	105 (36.2%)		r	
Yes	107 (27%)	42 (39.3%)	0.58	1.14	(0.72-1.79)

r: reference category; CI: confidence interval.

**Table 2:** Depression among type-II diabetic patients (*N* = 397) in relation to their clinical data.

Characteristic	Total no (%)	Depression no (%)	*P* value	Odds ratio	95% CI
Clinical data:					
Family history of DM:					
No	233 (58.7%)	79 (33.9%)		r	
Yes	164 (41.3%)	68 (41.5%)	0.12	1.38	(0.91-2.08)
Duration of DM:					
<5 y	157 (39.6%)	24 (15.3%)		r	
5-9 y	197 (49.6%)	101 (51.3%)	0.0001	5.83	(3.48-9.77)
10+	43 (10.8%)	22 (51.2%)	0.0001	5.80	(2.78-12.15)
Type of treatment:					
Oral hypoglycemic drugs	179 (45.1%)	29 (16.2%)		r	
Insulin only	47 (11.8%)	21 (44.7%)	0.0001	4.18	(2.07-8.41)
Both	171 (43.1%)	97 (56.7%)	0.0001	6.78	(4.11-11.17)
Compliance to diet:					
No	175 (44.1%)	80 (45.7%)	0.001	1.95	(1.28-2.94)
Yes	222 (55.9%)	67 (30.2%)		r	
Compliance to physical activity:					
No	218 (54.9%)	104 (47.7%)	<.0001	2.88	(1.86-4.45)
Yes	179 (45.1%)	43 (24%)		r	
Compliance to medications:					
No	65 (16.4%)	25 (38.5%)	0.79	1.07	(0.62-1.85)
Yes	332 (83.6%)	122 (36.7%)		r	
Compliance to regular foot examination:					
No	187 (47.1%)	73 (39%)	0.43	1.17	(0.78-1.77)
Yes	210 (52.9%)	74 (35.2%)		r	
Compliance to daily blood sugar measurement:					
No	290 (73%)	114 (39.3%)	0.12	1.45	(0.90-2.33)
Yes	107 (27%)	33 (30.8%)		r	
Complications of DM:					
Retinopathy					
No	311 (78.3%)	107 (34.4%)		r	
Yes	86 (21.7%)	40 (46.5%)	0.039	1.66	(1.02-2.69)
Nephropathy					
No	371 (93.5%)	132 (35.6%)		r	
Yes	26 (6.5%)	15 (57.7%)	0.023	2.47	(1.10-5.53)
Neuropathy					
No	338 (85.1%)	107 (31.7%)		r	
Yes	59 (14.9%)	40 (67.8%)	<.0001	4.55	(2.51-8.22)
Libido					
No	331 (83.4%)	100 (30.2%)		r	
Yes	66 (16.6%)	47 (71.2%)	0.0001	5.71	(3.19-10.22)
Family history of depression					
No	383 (96.5%)	138 (36%)		r	
Yes	14 (3.5)	9 (64.3)	0.03	3.19	(1.05-9.72)

r: reference category; CI: confidence interval.

**Table 3:** Predictors of depression among studied type-II diabetic patients (*n* = 397).

Predictors	B	S.E.	Sig.	Exp (B)	95% CI for EXP (B)	
					Lower	Upper
Family income	-.694	.268	.009	.500	.296	.844
Duration of DM	.861	.203	.000	2.367	1.590	3.523
Compliance to physical activity	-.947	.256	.000	.388	.235	.641
Neuropathy	1.981	.486	.000	7.248	2.794	18.802
Libido	1.546	.327	.000	4.695	2.473	8.911
Constant	-.418	.660	.527	.658		

## Data Availability

Data analyzed in this study are available on request to the corresponding author.
